# Case report: abdominal cocoon syndrome following peritoneal dialysis presenting as closed-loop small bowel obstruction

**DOI:** 10.1093/jscr/rjag737

**Published:** 2026-08-24

**Authors:** Islam Bassam Atawna, Bessan H Dababseh, Ala’a S Ghnimat, Lubna W AbuHamdiya, Abdallah H Dababsseh, Sujood Abudaia, Mohammed Maraqa

**Affiliations:** Faculty of Medicine, Palestine Polytechnic University, Wadi Al-Haria, PO Box 198, Hebron, Palestine; Faculty of Medicine, Palestine Polytechnic University, Wadi Al-Haria, PO Box 198, Hebron, Palestine; Faculty of Medicine, Palestine Polytechnic University, Wadi Al-Haria, PO Box 198, Hebron, Palestine; Faculty of Medicine, Palestine Polytechnic University, Wadi Al-Haria, PO Box 198, Hebron, Palestine; Faculty of Medicine, Palestine Polytechnic University, Wadi Al-Haria, PO Box 198, Hebron, Palestine; Ain Shams University, 38 Abbassia Square, Cairo 1181, Egypt; Faculty of Medicine, Palestine Polytechnic University, Wadi Al-Haria, PO Box 198, Hebron, Palestine; Surgical Department, Al Ahli Hospital, Farsh Al-Hawa, Hebron, Palestine

**Keywords:** encapsulating peritoneal sclerosis, abdominal cocoon, peritoneal dialysis, small bowel obstruction

## Abstract

Encapsulating peritoneal sclerosis (EPS) is a rare but life-threatening complication of long-term peritoneal dialysis (PD) characterized by fibrocollagenous encapsulation of the bowel. We report a 37-year-old female with a 5-year history of PD who presented with acute abdominal pain, bilious vomiting, and abdominal distension secondary to a closed-loop small bowel obstruction. Computed tomography (CT) demonstrated characteristic findings, including bowel tethering, peritoneal thickening, and septated fluid collections suggestive of EPS. Emergency exploratory laparotomy confirmed extensive bowel encapsulation by a dense fibrotic membrane. The patient underwent enterolysis, adhesiolysis, excision of the fibrotic capsule, bowel resection, and primary anastomosis. The patient gradually recovered and was transitioned permanently to hemodialysis. This case emphasizes the diagnostic challenge of EPS and highlights the essential role of CT and timely surgical intervention in reducing the significant morbidity and mortality associated with this condition.

## Introduction

Peritoneal dialysis (PD) is a widely used home-based therapy for end-stage renal disease (ESRD), with 50%–80% of patients developing peritoneal fibrosis within the first year [1]. Encapsulating peritoneal sclerosis (EPS) is a rare but severe complication of long-term PD, with an incidence of 0.7%–3.3% and a reported case fatality rate of ~50% [1–3]. It is characterized by progressive fibrocollagenous encasement of the small bowel, leading to recurrent intestinal obstruction and malnutrition. Clinical presentation is often non-specific, including abdominal pain, distension, and altered bowel habits, which frequently results in delayed diagnosis [4]. No specific laboratory marker is available, and computed tomography (CT) remains the imaging modality of choice, typically demonstrating peritoneal thickening, tethered bowel loops, and septated fluid collections; however, many cases are only definitively diagnosed intraoperatively [3, 4]. We report a case of EPS presenting as acute closed-loop obstruction to highlight diagnostic challenges and emphasize the importance of early CT imaging and timely surgical intervention.

## Case presentation

A 37-year-old female presented with sudden-onset progressive epigastric pain radiating to the back, bilious vomiting, chills, and abdominal distension, without prior similar episodes.

She had ESRD (right renal atrophy, diagnosed at age 30), managed with PD for 5 years before transitioning to hemodialysis (three sessions/week). Comorbidities included hypertension (doxazosin, carvedilol) and hypothyroidism (levothyroxine). Surgical history comprised PD catheter insertion/removal, two arteriovenous fistulae (radiocephalic and brachiocephalic), umbilical hernia repair, bilateral cataract surgery, and pigtail catheter insertion for ascites. She was allergic to penicillin and vancomycin.

**Figure 1:**
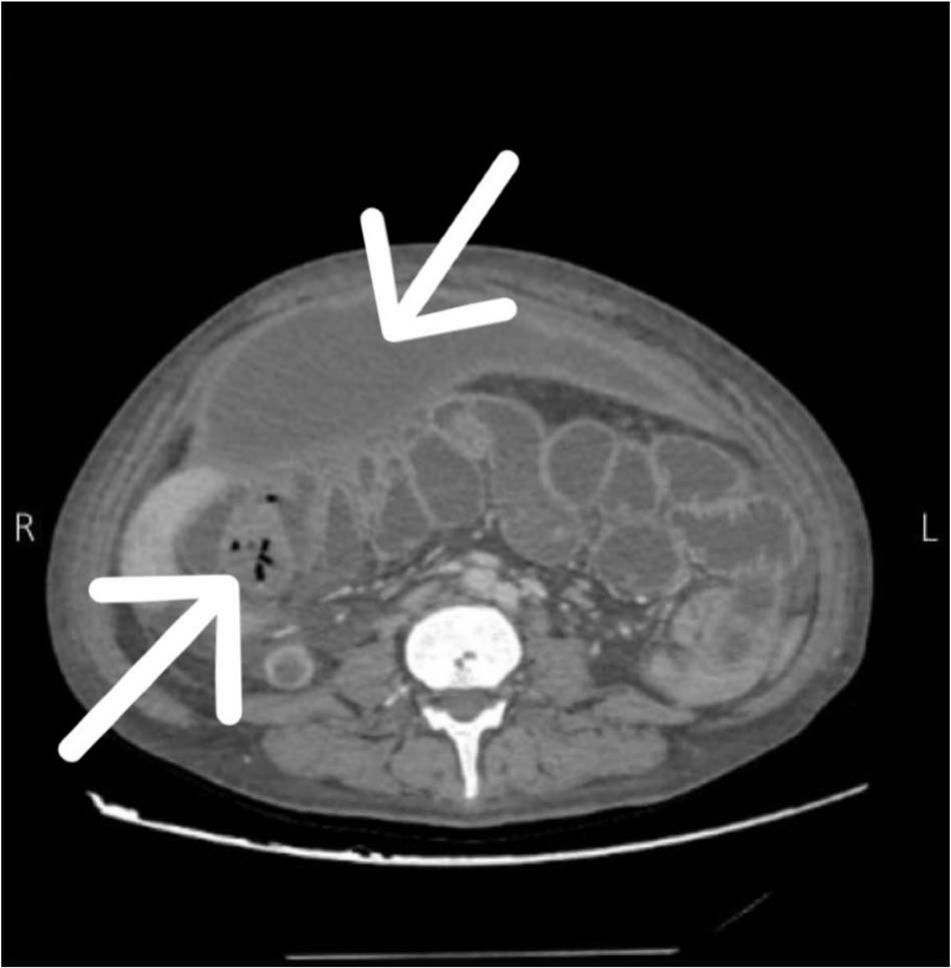
Contrast-enhanced CT scan of the abdomen; the upper arrow highlights diffuse peritoneal thickening characteristic of EPS, while the lower arrow indicates the specific site of the acute “closed-loop” intestinal obstruction.

**Figure 2:**
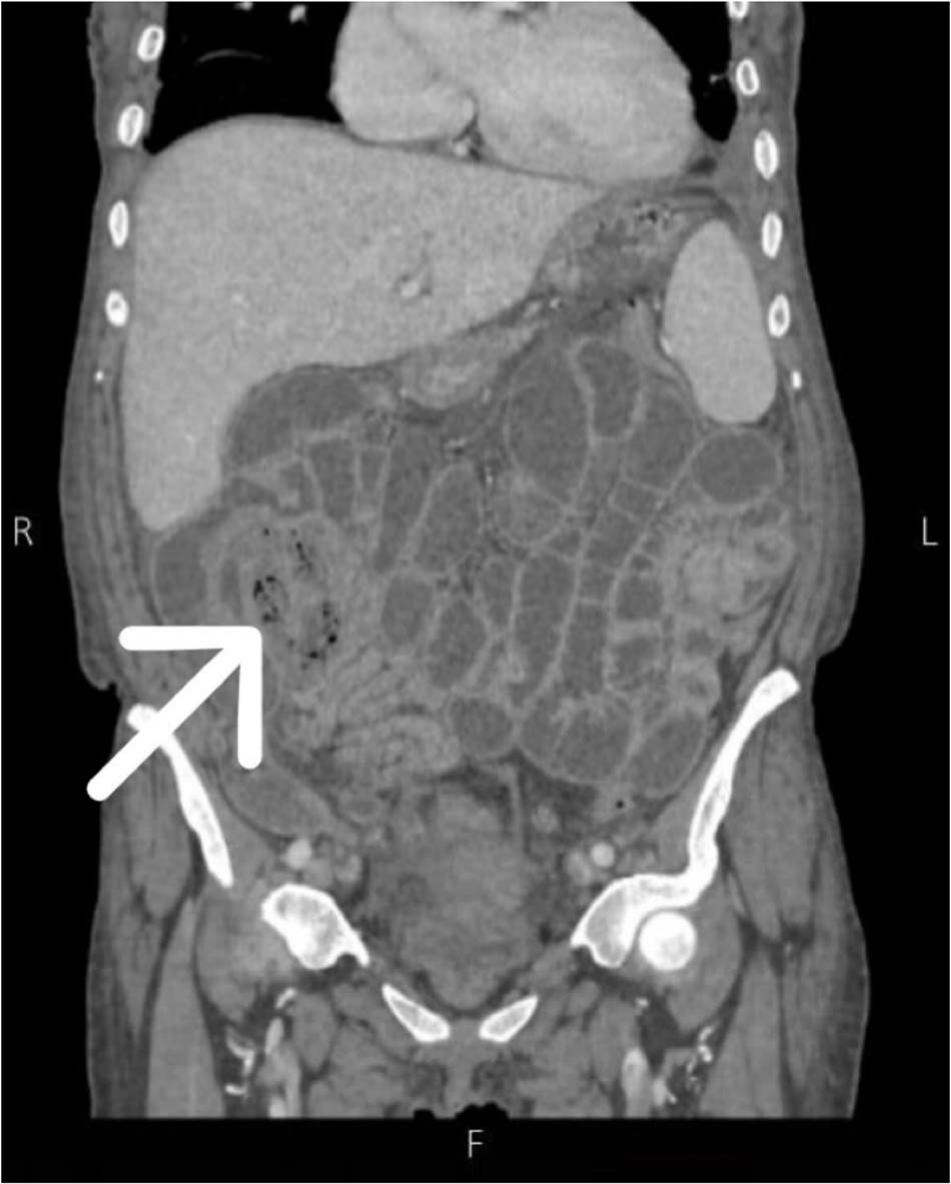
Detailed CT view focusing on the transition point; the arrow indicates the site of obstruction, clearly showing significant proximal bowel dilation (pre-stenotic) and distal bowel collapse, which are hallmark signs of a complete mechanical obstruction.

Physical examination revealed a tense, distended abdomen with diffuse dullness to percussion. Axial CT demonstrated small bowel obstruction (Fig. 1); coronal imaging demonstrated centrally congregated bowel loops consistent with encapsulation (Fig. 2).

The patient underwent an emergency exploratory laparotomy. The small bowel was entirely encased within a dense fibrotic membrane with extensive inter-loop adhesions, confirming EPS. Surgical management included enterolysis, adhesiolysis, fibrotic capsule excision, resection of nonviable bowel with primary anastomosis, and abdominal wall collection drainage. She received postoperative care in the intensive care unit with intravenous fluids, blood products, analgesia, and antibiotics, recovering gradually. She was discharged on permanent hemodialysis with definitive discontinuation of PD.

Unfortunately, no additional long-term follow-up data were available beyond the patient’s hospital discharge.

## Discussion

EPS, also known as abdominal cocoon syndrome, is a rare but severe complication of long-term PD [5, 6]. It typically presents in a subacute manner with progressive malnutrition and partial bowel obstruction, whereas our patient developed an acute complete closed-loop obstruction after ~5 years of PD, within the recognized high-risk period [7–9].

The patient had a history of recurrent left-sided pleural effusions and episodic abdominal pain for nearly 2 years before the acute event. These may represent early inflammatory changes of peritoneal injury and fibrosis. Although non-specific, such findings in long-term PD patients should raise suspicion for evolving EPS rather than being attributed solely to dialysis-related complications [6, 10].

The diagnosis is often delayed because of non-specific symptoms that mimic peritonitis, gastroparesis, or other gastrointestinal conditions, particularly among non-specialist clinicians [11, 12]. In this case, CT imaging played a pivotal role in establishing the diagnosis, although the full extent of the disease was only appreciated intraoperatively.

Medical therapy, including tamoxifen, corticosteroids, and immunosuppressants, may be useful in early inflammatory stages to slow progression [5, 8]. However, once acute obstruction occurs, urgent surgical intervention is required, highlighting the importance of early recognition before irreversible fibrosis develops.

Compared with previously reported cases of EPS-related bowel obstruction, our patient’s presentation is notable in two respects. First, rather than the classical subacute or partial obstructive course most commonly described, she developed a complete closed-loop obstruction requiring emergency surgery—a course typically associated with advanced, diffuse EPS rather than the localized or segmental variants that have been described as isolated transition-point obstructions occasionally amenable to initial conservative management [13]. Second, intraoperative findings confirmed diffuse total encapsulation of the small bowel, distinguishing this case from these localized forms and illustrating that the clinical spectrum of EPS ranges from segmental, transition-point disease to complete cocooning presenting as a surgical emergency. This distinction carries practical weight: while localized EPS may occasionally be managed conservatively with bowel rest and transition to hemodialysis [13], our case reinforces that once complete bowel encasement and closed-loop obstruction develop, prompt surgical exploration is mandatory regardless of prior medical therapy.

## Conclusion

EPS is a rare but potentially life-threatening complication of PD. Atypical early features—recurrent pleural effusions and intermittent abdominal pain—may precede overt obstruction by years. In patients with ˃5 years of PD, a low threshold for obtaining CT imaging, proactive transition to hemodialysis, and timely surgical intervention are essential to prevent catastrophic mechanical obstruction and reduce morbidity and mortality.

## References

[1] Patel SM, Patel VM, Patel SV et al. Encapsulating peritoneal sclerosis leading to small bowel obstruction in a young woman with end-stage renal disease: a case report. Am J Case Rep 2024;25:1–4. 10.12659/AJCR.945398 PMC11530928 39462886

[2] de Faria Barros A, Moraes C, Pinto MBS et al. Is there an association between acyl-ghrelin and inflammation in hemodialysis patients? J Bras Nefrol 2013;35:120–6. 10.5935/0101-2800.20130020 23812569

[3] Ma’rifat RA, Suraharta IIJ IM. Encapsulating Peritoneal Sclerosis [Internet], Vol. 2. Treasure Island (FL): StatPearls Publishing, 2024, 306–12.

[4] South SC, Shah AP, Hartigan JR. Intraoperative diagnosis and surgical management of encapsulating peritoneal sclerosis: a case report. Cureus 2024;16:14–7. 10.7759/cureus.64515 PMC11321135 39139349

[5] Jagirdar RM, Bozikas A, Zarogiannis SG et al. Encapsulating peritoneal sclerosis: pathophysiology and current treatment options. Int J Mol Sci 2019;20:1–21. 10.3390/ijms20225765 PMC6887950 31744097

[6] de Sousa E, del Peso-Gilsanz G, Bajo-Rubio MA et al. Encapsulating peritoneal sclerosis in peritoneal dialysis. A review and European initiative for approaching a serious and rare disease. Nefrologia 2012;32:707–14. 10.3265/Nefrologia.pre2012.Jul.11615 23169353

[7] Chien CY, Tsai MT, Chen JY et al. Encapsulating peritoneal sclerosis outcomes: a retrospective study on the significance of nutritional parameter variations. BMC Nephrol 2025;26:509. 10.1186/s12882-025-04111-5 40890645 PMC12403267

[8] Hong KD, Bae JH, Jang YJ et al. Encapsulating peritoneal sclerosis: case series from a university center. Korean J Intern Med 2013;28:587–93. 10.3904/kjim.2013.28.5.587 24009455 PMC3759765

[9] Petrie MC, Traynor JP, MacTier RA. Incidence and outcome of encapsulating peritoneal sclerosis. Clin Kidney J 2016;9:624–9. 10.1093/ckj/sfw051 27478609 PMC4957727

[10] Li D, Li Y, Zeng H et al. Risk factors for encapsulating peritoneal sclerosis in patients undergoing peritoneal dialysis: a meta-analysis. PLoS One 2022;17:1–15. 10.1371/journal.pone.0265584 PMC8936465 35312717

[11] Akbulut S . Accurate definition and management of idiopathic sclerosing encapsulating peritonitis. World J Gastroenterol 2015;21:675–87. 10.3748/wjg.v21.i2.675 25593498 PMC4292304

[12] Mehmood M, Mirza AA, Sree GS et al. Abdominal cocoon syndrome. A rare cause of mechanical intestinal obstruction: a case report. Int J Surg Case Rep 2023;103:986–9. 10.1016/j.ijscr.2023.107875 PMC9876940 36682283

[13] Ghazan-Shahi S, Bargman JM. Case report: acute bowel obstruction with an isolated transition point in peritoneal dialysis patients; a presentation of encapsulating peritoneal sclerosis? BMC Nephrol 2016;17:1. 10.1186/s12882-015-0214-2 26727891 PMC4700655

